# Multimodal assessment of white matter tracts in amyotrophic lateral sclerosis

**DOI:** 10.1371/journal.pone.0178371

**Published:** 2017-06-02

**Authors:** Florian Borsodi, Valeriu Culea, Christian Langkammer, Michael Khalil, Lukas Pirpamer, Stefan Quasthoff, Christian Enzinger, Reinhold Schmidt, Franz Fazekas, Stefan Ropele

**Affiliations:** 1 Department of Neurology, Medical University of Graz, Graz, Austria; 2 Department of Radiology, Division of Neuroradiology, Medical University of Graz, Graz, Austria; Cornell University, UNITED STATES

## Abstract

Several quantitative magnetic resonance imaging (MRI) techniques have been proposed to investigate microstructural tissue changes in amyotrophic lateral sclerosis (ALS) including diffusion tensor imaging (DTI), magnetization transfer imaging, and R_2_* mapping. Here, in this study, we compared these techniques with regard to their capability for detecting ALS related white matter (WM) changes in the brain and their association with clinical findings. We examined 27 ALS patients and 35 age-matched healthy controls. MRI was performed at 3T, after which we analyzed the diffusion properties, the magnetization transfer ratio (MTR), and the effective transversal relaxation rate R_2_* in 18 WM tracts that were obtained by a fully automated segmentation technique. ALS patients, especially with a bulbar onset, showed a bilateral increase in radial and mean diffusivity, as well as a reduction in fractional anisotropy of the corticospinal tract (CST), and diffusion changes in the parietal and temporal superior longitudinal fasciculus. A reduction of the MTR was found in both CSTs and an R_2_* reduction was seen only in the left CST. Tract-specific diffusion properties were not related to clinical status in a cross-sectional manner but demonstrated some association with disease progression over three subsequent months. DTI reveals more widespread WM tissue changes than MTR and R_2_*. These changes are not restricted to the CST, but affect also other WM tracts (especially in patients with bulbar onset), and are associated with the short term course of the disease.

## Introduction

Amyotrophic lateral sclerosis (ALS) is a rapidly progressive and invariably fatal disorder of the motor neuron system, which characteristically affects both upper and lower motor neurons [1]. According to the revised El Escorial criteria, ALS diagnosis is based on specific clinical and electrophysiological findings [2]. In the early stage of the disease, however, these may still be subtle and thus provide only limited diagnostic certainty and prognostic information. Additional insights may come from quantitative assessment of the severity of damage to the corticospinal tract (CST), which is a pathologic hallmark feature of ALS besides the loss of motor neurons in the anterior horn. Several magnetic resonance imaging (MRI) techniques have already been applied to identify distinct morphological and structural changes in ALS [3,4]. So far, most quantitative MRI studies have been performed with various tract-based diffusion tensor imaging (DTI) techniques [5–8]. These attempts have been paralleled by a limited number of magnetization transfer imaging (MTI) studies [7,9]. More recently, there have also been reports on elevated iron levels in the motor cortex [10], as well as changes in R_2_* in the CST [11]. Post mortem analyses of ALS brains indicate that large areas of the white matter (WM), beyond the CST, may be affected by the disease, as well [12,13]. However, the extent to which pathological changes in WM tracts outside the CST may contribute to the clinical presentation and course of ALS is still largely unresolved. Apart from other techniques to assess ALS pathology DTI, MTI, and R_2_* mapping are now readily available on clinical MR scanners and require comparable acquisition time. It has not yet been investigated, however, how these imaging modalities compare in probing ALS related abnormalities in the same patient.

Therefore, this study aimed for a comprehensive assessment of microstructural tissue changes in ALS, both in a technical and a tract-related manner. The different contributions of tract-specific DTI, MTI analyses and iron mapping were compared with regards to their ability of defining ALS pathologies and we assessed the relation of observed abnormalities with disease progression. Furthermore, the presence of tract-specific abnormalities, beyond the CST, was also investigated by using a new probabilistic tractography approach.

## Materials and methods

### Subjects

In this prospective study we recruited 46 patients with suspected ALS over a 6-year period who were willing to undergo a comprehensive diagnostic work-up, including MRI of the brain and spinal cord, CSF examination and neurophysiologic assessment by an expert in neuromuscular diseases. During follow-up 15 patients were found to have other neuromuscular disorders. Furthermore, four patients had to be excluded due to inadequate image quality or lack of at least one 3-month clinical follow-up after the baseline MRI examination. From the remaining 27 patients, 13 were initially classified as possible and 14 as probable ALS according to the El Escorial criteria [2]. Furthermore, patients with already definite ALS classification were not included in this study. The overall severity of functional disturbances was described using the revised amyotrophic lateral sclerosis functional rating scale (ALSFRS-R), which consists of 13 items with a score ranging from 48 (best) to 0 points (worst) [14]. From this assessment at baseline and the duration of the disease, we calculated a disease progression rate R_DP_ [5] as follows:

$${\text{R}}_{\text{DP}}\;=\;\frac{48-\text{ALSFRS-R}}{{\text{T}}_{\text{disease}}(\text{mo})}.$$ (1)

The severity of upper motor neuron (UMN) impairment was described with the Penn UMN score, which contains of a bulbar segment and one segment for each limb, and ranges between 0 (best) and 32 (worst) [15].

For inclusion all patients also had to have at least one 3-month clinical follow-up after the baseline MRI examination, where the functional rating scale was re-assessed (ALSFRS-R_3M_). From these two rating scores, an ALSFRS-R progression rate, ΔALSFRS-R, was calculated as follows:

$$\Delta \text{ALSFRS}-\text{R}=\frac{{\text{ALSFRS-R}}_{\text{3M}}{-\text{ALSFRS-R}}_{{}_{baseline}}}{{\text{ALSFRS-R}}_{baseline}}100$$ (2)

Patients with a higher degree of disability often had problems to tolerate the rather long study protocol. In several cases, the MRI technician had to abort scanning when motion induced artifacts become severe or when patients requested an abort due to claustrophobia. It therefore was not possible to perform the full MRI protocol in all patients, which led to an unequal number of MTI and R_2_* datasets compared to DTI. None of the patients had clinical evidence for dementia.

Thirty-five healthy subjects, with a similar distribution of age and sex to ALS patients, who participated in an ongoing community-dwelling study on aging served as controls [16].

The study was approved by the ethics committee of the Medical University of Graz and signed consents were given by all participants.

### Acquisition of MRI data

Initially, all subjects underwent MRI of the brain on a 3 Tesla scanner (Siemens Healthcare, Tim Trio, Erlangen Germany) using a 12-channel head coil array. A T_1_-weighted 3D MPRAGE with 1 mm isotropic resolution (TR/TE/TI/flip angle = 1.9 s/2.15 ms/900 ms/9° and FoV = 256 mm) was used to obtain structural images. Additionally, an axial FLAIR sequence (TR/TE/TI = 10 s/69 ms/2.5 s, image resolution = 0.9x0.9x3 mm^3^) and T_2_-weighted fast spin echo sequence were performed to rule out brain lesions that could affect regional analyses.

DTI data were acquired with a 2D diffusion-weighted spin-echo sequence with a single shot EPI readout (TR/TE = 6700 ms/95 ms and NEX = 4) with a GRAPPA acceleration factor of 2, and an image resolution of 2x2x3 mm^3^. Twelve independent diffusion sensitizing directions and two b-values (b = 0 and 1000 s/mm^2^) were performed for subsequent reconstruction of the diffusion tensor images.

To map the magnetization transfer ratio (MTR), a spoiled 3D gradient echo sequence (TR/TE/flip angle = 40 ms/7.38 ms/15°, image resolution = 0.9x0.9x3 mm^3^) was executed with and without a Gaussian-shaped saturation pulse.

Mapping of the effective transversal relaxation rate R_2_* was done with a 3D spoiled gradient echo sequence (TR/TE_first_/flip angle = 35 ms/4.92 ms/15°) with 6 equally spaced echoes (bipolar readout gradient with an inter-echo spacing of 4.92 ms, in plane resolution = 0.9x0.9 mm^2^, slice thickness = 2 mm, FOV = 230 mm).

### Image analysis

**Fig 1:**
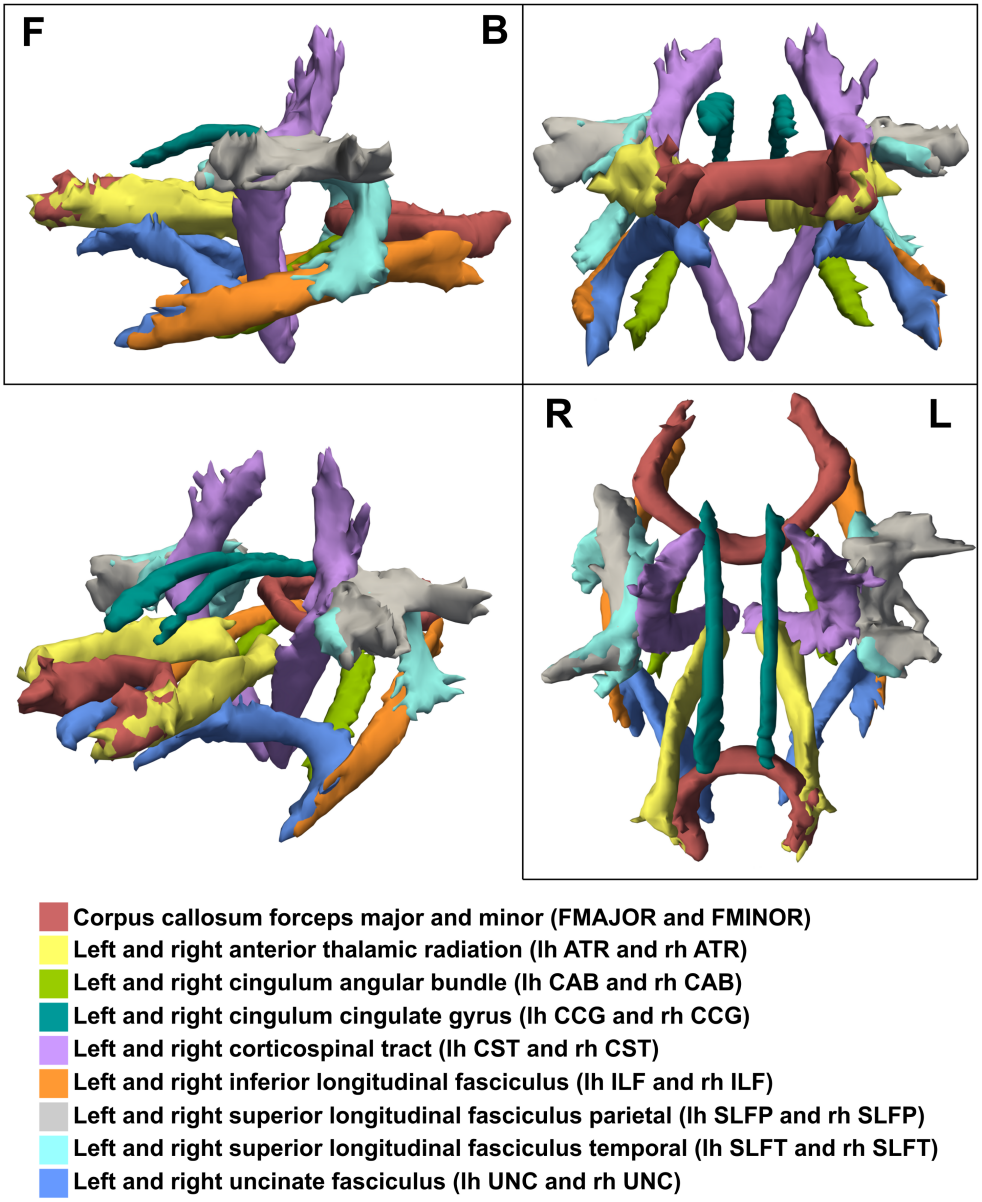
Tractography of a single ALS patient obtained by TRACULA with tract description. Shown are side- (F for front, B for back), front- and top- (R for right, L for left) as well as 3D views.

Diffusion-weighted images were visually reviewed to prevent results being affected by motion artifacts. Global probabilistic tractography and diffusion parameter analysis were performed using TRACULA [17], which is part of the FreeSurfer 5.3 [18] software package. TRACULA allows for automatic identification and reconstruction of 18 major WM tracts (Fig 1) based on the high resolution T_1_-weighted scan. It further determines all scalar parameters of the diffusion tensor (axial diffusivity AD, radial diffusivity RD, mean diffusivity MD and fractional anisotropy FA) for all tracts. To compensate for eddy currents, diffusion-weighted images were registered to a reference image without diffusion weighting. DTI and results of automated tractography were visually verified for correct calculation and reconstruction for every subject.

MTR maps were calculated from two images performed with (S_s_) and without (S_0_) an MT saturation pre-pulse, according to the formula [19]:

$$\text{MTR }=\;\frac{{\text{S}}_{0}-{\text{S}}_{\text{s}}}{{\text{S}}_{0}}.$$ (3)

For R_2_* mapping, all magnitude images were registered to the first echo to correct for image shifts evoked by the bipolar readout gradients. R_2_* maps were then determined by voxel-wise fitting of a mono-exponential decay function to the multi-echo data and considering non-Gaussian distributed noise.

**Fig 2:**
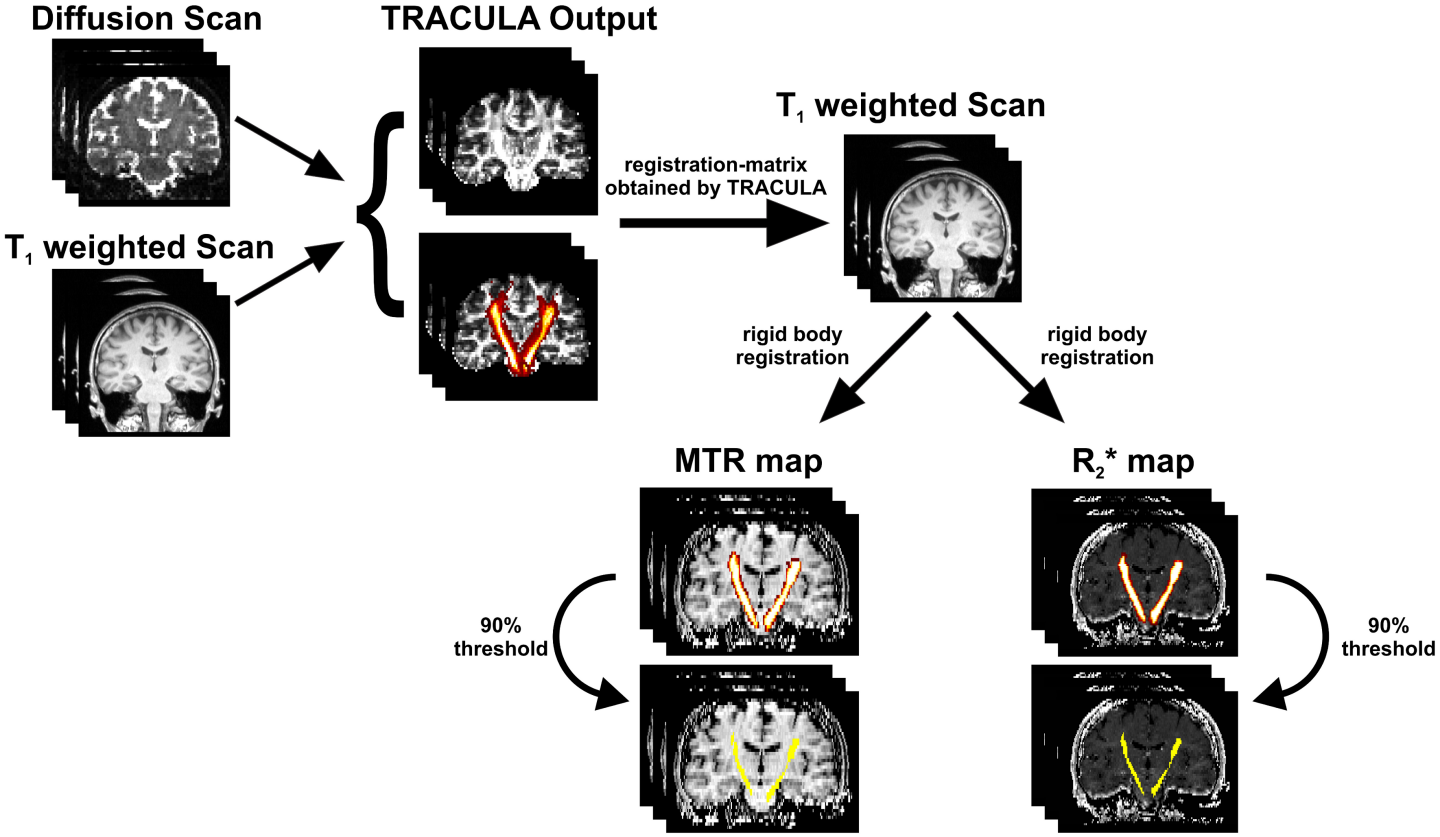
Tract processing pipeline. Based on the diffusion and T_1_ weighted scans, 18 WM tracts were reconstructed by TRACULA. Probability distribution maps of all tracts were reduced and binarized at 20% of their maximum value and then transformed into MTR and R_2_* Map space via registration-matrix (obtained by TRACULA) and rigid body registration. To prevent transformed tracts penetrating undesired regions, a conservative approach was deployed by applying a threshold of 90% of their maximum value.

For a corresponding analysis of MTR and R_2_* maps, all tracts were transformed into the MTR and R_2_* spaces (Fig 2). Following a conservative approach, probability distribution maps of each tract were reduced and binarized at 20% of their maximum value. Rigid body registration was done with FSL [20] on the high resolution T_1_-weighted image.

### Statistical analysis

All statistical analyses were performed with STATISTICA 7.1 (StatSoft, Tulsa, OK, USA). The Kolmogorov-Smirnov test was used to verify for normal distribution. A Student t-test or a Mann-Whitney U test, when appropriate, was applied to investigate regional differences in quantitative MRI measures between ALS patients and controls. Univariate linear regression analyses served to analyze the relation between diffusion properties, MTR, R_2_*, and clinical variables. A p value less than 0.05 was considered as statistically significant.

## Results

Overall, we analyzed the data of 27 patients of whom 16 had a bulbar and 11 a limb onset ALS. The demographic and clinical information on these patients is summarized in Table 1. The mean ALSFRS-R at baseline was 38.8 ± 8.7, and patients with limb onset showed a higher, i.e. better, ALSFRS-R compared to those with bulbar onset. This was also reflected in the R_DP_ with a mean R_DP_ of 0.8 ± 1.1 for the entire group. Over the first 3-months clinical follow-up, the entire patient group and both subgroups showed a deterioration of the ALSFRS-R compared to baseline.

**Table 1:** Demographic and clinical data of cohort.

Group	Sex		Age^†^ (y)	Disease Duration at MRI^†^ (mo)	ALSFRS-R^†^	ALSFRS-R_3M_^†^	R_DP_^†^
	F	M					
**ALS**	10	17	58.1±12.3	16.1±12.7	38.8±8.7	36.3±8.8	0.8±1.1
**ALS bulbar**	8	8	59.8±11.8	16.6±13.9	35.9±10.0	33.1±9.8	1.1±1.4
**ALS limb**	2	9	55.6±13.2	15.3±11.3	43.0±3.5	41.1±4.3	0.8±0.4
**Controls**	12	23	57.7±13.0	-	-	-	-

^†^ Data are means ± standard deviation,

ALS = amyotrophic lateral sclerosis, F = female, M = male, y = years, mo = months, ALSFRS-R = revised ALS functional rating scale, ALSFRS-R_3M_ = ALSFRS-R 3 months follow-up, R_DP_ = disease progression rate

### Diffusion tensor imaging

ALS related changes of diffusion properties (AD, RD, MD, FA) are listed in Table 2. AD remained unchanged in ALS. However, a significant increase of RD and MD, along with a concomitant decrease of FA, was observed in both the left and right CST. A similar pattern of diffusional abnormalities was also found in the left superior longitudinal fasciculus parietal (SLFP), and minor abnormalities were seen in the right SLFP and in the superior longitudinal fasciculus temporal (SLFT) bilaterally. No other tracts showed abnormal diffusion properties.

**Table 2:** Group analysis results of quantitative MRI in 18 WM tracts for whole cohort. Provided are T- or Z-values.

	AD	RD	MD	FA	MTR	R_2_*
	T	T	T	T	Z	Z
**N (controls/ALS)**	35/27	35/27	35/27	35/27	30/14	33/24
**FMAJOR**	0.38	-0.28	-0.01	0.30	-1.61	-0.78
**FMINOR**	0.81	1.41	1.36	-1.15	0.30	0.03
**lh ATR**	0.43	1.11	0.94	-1.21	-0.58	0.57
**lh CAB**	0.16	0.18	0.20	0.14	0.71	1.03
**lh CCG**	-0.46	1.33	1.05	-1.36	-1.18	0.87
**lh CST**	0.96	5.04**	4.64**	-3.94**	-2.62*	-1.29
**lh ILF**	0.60	0.52	0.67	-0.16	-1.59	0.95
**lh SLFP**	1.33	2.45*	2.17*	-2.66*	-1.08	-1.20
**lh SLFT**	1.37	1.91	1.96	-1.38	-0.66	-1.60
**lh UNC**	1.33	1.30	1.37	-0.91	-0.08	-0.40
**rh ATR**	0.27	1.66	1.27	-1.98	-1.01	-0.58
**rh CAB**	0.48	0.80	0.82	-0.27	-0.55	0.44
**rh CCG**	0.43	1.39	1.43	-1.08	-0.42	0.44
**rh CST**	0.21	4.56**	3.55**	-3.96**	-3.04**	-0.18
**rh ILF**	-0.21	0.63	0.39	-0.77	0.08	-0.10
**rh SLFP**	0.33	1.72	1.33	-2.34*	-0.55	0.71
**rh SLFT**	1.77	1.78	2.08*	-0.83	-0.66	0.48
**rh UNC**	-0.54	0.56	0.24	-1.16	0.79	-0.63

** p < 0.05 (after Bonferroni correction),

* p value < 0.05,

AD = axial diffusivity, RD = radial diffusivity, MD = mean diffusivity, FA = fractional anisotropy, MTR = magnetization transfer ratio, N = number of subjects, ALS = amyotrophic lateral sclerosis, FMAJOR = corpus callosum forceps major, FMINOR = corpus callosum forceps minor, ATR = anterior thalamic radiation, CAB = cingulum angular bundle, CCG = cingulum cingulate gyrus, CST = corticospinal tract, ILF = inferior longitudinal fasciculus, SLFP = superior longitudinal fasciculus parietal, SLFT = superior longitudinal fasciculus temporal, UNC = uncinate fasciculus, lh = left hemisphere, rh = right hemisphere

When considering bulbar and limb onset separately, diffusion properties showed specific differences, which are summarized in Tables 3 and 4. Patients with a bulbar onset had diffusion properties which were in line with findings for the entire ALS cohort, but also showed an increase in AD in the left uncinate fasciculus (UNC). In contrast, patients with a limb onset showed altered diffusion properties in the CSTs only.

**Table 3:** Tract-specific abnormalities in patients with bulbar onset vs. controls.

	AD	RD	MD	FA	MTR	R_2_*
	Z	Z	Z	Z	Z	Z
**N (controls/ALS)**	35/16	35/16	35/16	35/16	30/9	33/13
**FMAJOR**	0.02	0.08	0.18	0.08	-0.70	-0.70
**FMINOR**	0.77	1.66	1.54	-1.38	0.50	0.70
**lh ATR**	0.83	1.60	1.48	-1.54	-0.23	1.33
**lh CAB**	1.06	0.83	1.12	-0.02	0.57	1.52
**lh CCG**	0.00	1.89	1.54	-1.83	-1.00	0.62
**lh CST**	1.06	4.24**	3.98**	-3.35**	-3.10**	-2.09*
**lh ILF**	0.83	1.28	1.26	-0.87	-1.27	0.62
**lh SLFP**	1.52	2.48*	2.38*	-2.72*	-1.20	-1.16
**lh SLFT**	1.54	1.71	1.77	-0.99	-0.73	-1.16
**lh UNC**	2.03*	1.60	1.79	-1.20	1.53	-0.55
**rh ATR**	0.73	1.66	1.38	-2.33*	-0.37	-0.87
**rh CAB**	0.59	0.51	0.81	0.10	0.90	0.45
**rh CCG**	-0.20	1.64	1.24	-1.60	0.18	0.74
**rh CST**	0.08	3.82**	3.11**	-3.45**	-2.40*	-0.72
**rh ILF**	0.81	0.79	0.63	-0.53	-0.87	0.13
**rh SLFP**	1.36	1.89	1.81	-2.25*	-0.97	1.65
**rh SLFT**	1.44	2.05*	2.11*	-1.38	-1.27	1.48
**rh UNC**	-0.02	0.65	0.37	-1.06	0.15	-0.26

** p < 0.05 (after Bonferroni correction),

* p value < 0.05,

AD = axial diffusivity, RD = radial diffusivity, MD = mean diffusivity, FA = fractional anisotropy, MTR = magnetization transfer ratio, N = number of subjects, ALS = amyotrophic lateral sclerosis, FMAJOR = corpus callosum forceps major, FMINOR = corpus callosum forceps minor, ATR = anterior thalamic radiation, CAB = cingulum angular bundle, CCG = cingulum cingulate gyrus, CST = corticospinal tract, ILF = inferior longitudinal fasciculus, SLFP = superior longitudinal fasciculus parietal, SLFT = superior longitudinal fasciculus temporal, UNC = uncinate fasciculus, lh = left hemisphere, rh = right hemisphere

**Table 4:** Tract-specific abnormalities in patients with limb onset vs. controls.

	AD	RD	MD	FA	MTR	R_2_*
	Z	Z	Z	Z	Z	Z
**N (controls/ALS)**	35/11	35/11	35/11	35/11	30/5	33/11
**FMAJOR**	-0.06	-0.27	-0.45	0.27	-2.03*	-0.53
**FMINOR**	-0.73	-0.01	-0.76	-0.04	-0.14	-0.72
**lh ATR**	-0.12	-0.53	-0.37	0.53	-0.75	-0.53
**lh CAB**	-0.40	-0.19	-0.27	0.32	0.52	0.04
**lh CCG**	-0.71	0.27	-0.30	-0.35	-0.80	0.77
**lh CST**	-0.12	2.10*	1.97*	-1.97*	-0.52	0.15
**lh ILF**	0.09	-0.68	-0.40	0.86	-1.18	0.91
**lh SLFP**	-0.19	1.07	0.84	-0.97	-0.33	-0.72
**lh SLFT**	0.06	0.61	0.24	-0.73	-0.19	-1.40
**lh UNC**	-0.64	-0.45	-0.53	0.09	-2.31*	-0.07
**rh ATR**	0.35	0.37	0.53	-0.24	-1.37	-0.01
**rh CAB**	-0.42	-0.24	-0.12	-0.30	-2.31*	0.23
**rh CCG**	0.30	-0.24	0.45	0.61	-1.04	-0.09
**rh CST**	-0.35	2.74*	1.84	-2.18*	-2.29*	0.50
**rh ILF**	-1.48	-0.17	-0.68	-0.53	1.37	-0.31
**rh SLFP**	-0.84	0.71	0.30	-1.43	0.33	-0.64
**rh SLFT**	0.73	0.58	0.53	0.04	0.57	-0.83
**rh UNC**	-1.62	-0.17	-0.91	-0.66	1.27	-0.77

* p value < 0.05,

AD = axial diffusivity, RD = radial diffusivity, MD = mean diffusivity, FA = fractional anisotropy, MTR = magnetization transfer ratio, N = number of subjects, ALS = amyotrophic lateral sclerosis, FMAJOR = corpus callosum forceps major, FMINOR = corpus callosum forceps minor, ATR = anterior thalamic radiation, CAB = cingulum angular bundle, CCG = cingulum cingulate gyrus, CST = corticospinal tract, ILF = inferior longitudinal fasciculus, SLFP = superior longitudinal fasciculus parietal, SLFT = superior longitudinal fasciculus temporal, UNC = uncinate fasciculus, lh = left hemisphere, rh = right hemisphere

### Magnetization transfer and R_2_*

The MTR was assessed in a subset of 14 patients, including 9 with bulbar and 5 with limb onset, and 30 controls. Results from the nonparametric group analyses are shown in Tables 2–4. When considering all ALS patients, a reduced MTR was only found in the left and right CST. The same observation was made for patients with a bulbar onset (Table 3). In patients with a limb onset, a MTR reduction was found in corpus callosum forceps major (FMAJOR), left UNC, right cingulum angular bundle (CAB), and the right CST (Table 4). MTR in all other tracts was not different from controls.

R_2_* values were obtained from 24 ALS patients, including 13 with bulbar and 11 with limb onset, and 33 healthy controls. No differences in R_2_* were found between ALS patients and controls when considering all patients (Table 2). However, when considering only patients with a bulbar onset, a reduction of R_2_* was observed in the left CST (Table 3). A complete data set including DTI, MTI, and R_2_* mapping was available on 14 ALS patients and 28 controls. Nonparametric analysis of this subgroup yielded similar results regarding the contribution of the individual techniques as when using all available patient data (see online supplemental S1 Table). DTI showed changes in the FMAJOR, the left cingulum cingulate gyrus (CCG), left SLFP and left SLFT, as well as in bilateral tracts of the CST. MTR showed changes only in the bilateral tracts of the CST and R_2_* showed no differences between ALS patients and controls. Thus most widespread abnormalities were observed with DTI.

### Clinical correlations

The estimated R_DP_ was not associated with any quantitative MRI values. Linear regression analyses also showed very few associations of quantitative MRI with disease duration and ALS severity in a cross-sectional manner, but some relation was seen with disease progression as expressed by the change in the ALSFRS-R over three months. This relationship was not only observed for diseased tracts but also in some tracts that appeared normal when compared to healthy controls. Associations were mainly seen with DTI metrics rather than MTR and R_2_*. The mean Penn UMN score was 5.2 ± 4.4 and was related to the R_2_* of the left and right CST and to the MTR of the FMAJOR. The results of the linear regression analyses are listed in the online S2 Table, where only tracts with significant associations are shown.

## Discussion

This study confirms and extends previous work on the individual contribution of quantitative MR techniques including DTI, MTR and R_2_* mapping to capture ALS pathology. In contrast to previous reports, this study relied on a probabilistic fiber tracking approach that allowed to perform unbiased analyses in 18 WM tracts, including the CST. Gray matter structures, including the motor cortex were not considered in this study. Nonetheless, the most consistent finding for all techniques was the bilateral affection of the CST. This is in line with pathology and the general observation that the CST is impaired in ALS [6,8,21]. However, some studies showed impairment for only one side [7] or even failed to detect a significant damage to the CST [11]. Additionally, differences between patients and controls were found in complementary tracts of SLFP and SLFT. This observation was already made earlier [8] and lead to the suggestion that ALS extends to more widespread areas of the central nervous system. Some studies also found changes in the corpus callosum and the extramotor WM which further supports the assumption of a more widespread affection [22].

Part of such changes may also relate to cognitive deficits which are known to occur in up to 30% of patients with ALS [23]. When considering patients with a bulbar and limb onset separately, different findings have been reported for the respective subgroups [24,25]. While in patients with a limb onset ALS only the bilateral CST was significantly affected in our patients, a bulbar onset of ALS was associated with damage of multiple WM tracts. This is also in line with earlier observations [25,26] and the worse prognosis of such patients [1].

All analyses were based on an automated global probabilistic tractography approach [17], which was previously evaluated in ALS [8]. A major benefit of this approach is the exclusion of user bias from manual outlining of the region of interest. Another benefit is that all analyses can be performed on a subject level. In contrast to tract based spatial statistics (TBSS), the tracts or their skeletons are also not subject to misregistration and therefore can be more reliably assessed.

Our findings show that DTI provides a higher capability for detecting ALS-related changes of cerebral WM microstructure than MTR and R_2_* mapping. Earlier studies have mostly focused on FA and MD, and did not consider changes in RD and AD [6]. From other work into the relevance of these scalar properties of the diffusion tensor it has been suggested that RD was related to myelin changes [27] while AD was an indicator for axonal alterations [28]. Interestingly, and against our expectations, we found that RD but not AD was elevated in ALS patients. Although, the interpretation of the diffusion tensor needs some caution [29], this observation not only explains the reduction of FA and the increase of MD, but suggests damage to the myelin rather than to the axons as the prevailing morphologic substrate [6,12,30]. Overall, these findings imply that for future studies, a more detailed analysis of the diffusion tensor, like higher order tensor models or composite measurements, should be considered.

Consistent with previous reports [9,31], we also observed a reduction of the MTR in the CST of ALS patients. These findings were also reported earlier in a cross-sectional analysis of the CST in the spinal cord [32], as well as in motor-related areas and extra-motor areas of the cortex [33], However, our results are in conflict with other studies, where MTR was elevated in patients [7], or where no differences were found between ALS patients and controls [34]. These diverse findings might also be caused by different sequence protocols. Thus, MTR is a semi-quantitative value and dependent on multiple factors including TR, T_1_, flip angle, and tissue type [19]. Nonetheless, although MTR was assessed in a relative small sample of 14 patients, it was capable to detect significant changes in the CST. Even though the degree of changes was not as high as for diffusion parameters, the differences in MTR paralleled those in DTI. Since MTR is considered a marker for myelin [35] these findings provide further support for demyelination as an important component of ALS pathology consistent with the observed increase in RD.

R_2_* mapping has been rarely used in previous ALS studies. The motivation to include R_2_* mapping was driven by the observation that R_2_* was increased in the CST of ALS patients in a previous study [11] where it was speculated that this could be indicative of increased iron levels. More recent studies have also shown T_2_* and quantitative susceptibility changes [36,37] in the motor cortex of ALS patients which are most likely induced by focal iron accumulation. In contrast to this earlier study suggesting R_2_* increases in the CST of ALS we here performed a tract-wise analysis and found no R_2_* changes with the exception of the CST in the bulbar cohort which also exhibited a reduced MTR. In line with an increased RD, R_2_* shortening thus probably is a consequence of demyelination rather than due to elevated iron concentration. The interpretation of R_2_* changes in WM is usually difficult due to its dependency on fiber orientation and due to counteracting magnetic effects of iron and myelin [38]. However, increased iron deposition would have increased R_2_* and since the CST runs mainly parallel to the direction of the main magnetic field, orientational effects can therefore be neglected.

Reports of individual studies suggest correlations between DTI parameters and the ALSFRS-R [14,21], disease duration and disease progression [30,39], and the UMN score [39,40]. In this study, we did not find a convincing relationship between diffusion properties and disease duration or severity which is disappointing. This indicates that a MRI snapshot of this rapidly progressing disease even when using quantitative techniques is not able to explain a patient’s clinical condition. However, DTI was associated with disease progression as expressed by the ΔALSFRS-R. Interestingly such relationship was also observed for WM tracts that appeared normal when compared to healthy controls. While MTR and R_2_* exhibited an overall smaller alteration, they were related to disease duration and the Penn UMN score, which was not the case for DTI. One could therefore hypothesize, that MTR and R_2_* reflect more chronic or accumulated damage, while DTI may be indicative of ongoing disease activity. In this context, it is also notable that the MTR in the forceps major was reduced compared to healthy controls and highly related to the Penn UMN score. DTI and MTR, therefore, seem to provide a complementary picture. In addition, it should be noted that here the UMN score was not used to address prognosis or survival but rather to find associations with motor deficits in ALS. Furthermore, it should be considered that the overall small sample size together with the clinical heterogeneity across the ALS patients studied may have contributed to the limited number of significant clinicoradiologic associations observed in this study.

Unfortunately, a main limitation of our study comes from the fact that we were not able to collect more long-term clinical follow-up data or any MRI follow-up scans. This should be a priority for future studies in ALS as indicated by observed associations of quantitative MRI metrics already with a three months clinical follow up. Furthermore, not all patients were able to undergo the full MRI protocol because of their advanced disability or psychological conditions which resulted in an unequal number of MTR and R_2_* datasets compared to DTI. This also led to partly very small patient numbers, e.g. only 5 patients with limb onset ALS had information on MTR which needs to be considered in the interpretation of the statistical results. However, analyzing only those patients who had completed the full MRI protocol led to a similar picture of differences as when using all patient data. Finally, although none of our patients had clinically evident dementia detailed neuropsychological testing was not performed to search for subtle coexisting cognitive deficits.

In conclusion, DTI appears to show more widespread WM abnormalities when probing ALS related changes than MTR and R_2_*. These changes are not restricted to the CST and seem to be more widespread, especially in patients with bulbar onset ALS. While DTI properties are not related to disease duration and clinical status, they could serve to predict disease progression at least on a short term basis. This needs confirmation in prolonged follow-up studies. MTR and R_2_* provide some complementary information but appear less capable for detecting ALS-related tissue changes.

## Supporting information

S1 Table Nonparametric group analysis results of quantitative MRI in 18 WM tracts for patients and controls with completed MRI protocol. Provided are T- or Z-values.
(PDF)

S2 Table Relationship between clinical status and quantitative MRI for individual tracts. The Pearson correlation coefficient is provided.
(PDF)

S3 Table Patients and controls raw data. (XLSX)
