# Supplementary material for: Multimodal assessment of white matter tracts in amyotrophic lateral sclerosis
Source: PLoS One. 2017 Jun 2;12(6):e0178371. doi: 10.1371/journal.pone.0178371 (PMC5456080; doi:10.1371/journal.pone.0178371)
Supplement: S1 Table — Provided are T- or Z-values. (PDF) [file pone.0178371.s001.pdf]

S1 Table. Nonparametric Group Analysis Results of Quantitative MRI in 18 WM Tracts for Patients and Controls with completed MRI protocol. Provided are T- or Z-values

	**AD**	**RD**	**MD**	**FA**	**MTR**	**R_2***
	**Z**	**Z**	**Z**	**Z**	**Z**	**Z**
**N (controls/ALS)**	28/14	28/14	28/14	28/14	28/14	28/14
**FMAJOR**	2.03*	0.29	1.47	0.56	-1.49	-0.24
**FMINOR**	0.56	1.71	1.31	-1.36	0.05	-0.77
**lh ATR**	1.52	0.99	1.44	-0.48	-0.59	0.16
**lh CAB**	0.80	0.48	0.88	0.11	0.80	0.45
**lh CCG**	-0.88	1.97*	1.07	-1.97*	-1.09	1.33
**lh CST**	0.99	3.58**	3.55**	-3.04**	-2.48*	-1.68
**lh ILF**	0.64	1.09	1.28	-0.51	-1.57	0.19
**lh SLFP**	1.49	2.35*	2.27*	-2.45*	-1.01	-1.01
**lh SLFT**	1.63	2.16*	2.24*	-1.41	-0.67	-0.88
**lh UNC**	1.23	0.56	0.80	0.08	-0.13	-1.52
**rh ATR**	1.17	1.55	1.44	-1.49	-1.09	-0.48
**rh CAB**	0.40	0.53	0.83	-0.27	-0.72	-0.75
**rh CCG**	-1.31	1.39	0.64	-1.92	-0.40	0.67
**rh CST**	-0.21	2.99*	2.43*	-2.69*	-3.06**	-1.09
**rh ILF**	0.43	1.20	1.17	-1.31	-0.11	0.27
**rh SLFP**	1.15	1.47	1.57	-1.55	-0.75	1.15
**rh SLFT**	0.96	1.87	1.92	-0.93	-0.88	0.75
**rh UNC**	0.29	0.88	0.56	-1.12	0.63	-1.15

** $p_{Bonferroni} < 0.05$, * p value < 0.05, AD = axial diffusivity, RD = radial diffusivity, MD = mean diffusivity, FA = fractional anisotropy, MTR = magnetization transfer ratio, N = number of subjects, ALS = amyotrophic lateral sclerosis, FMAJOR = corpus callosum forceps major, FMINOR = corpus callosum forceps minor, ATR = anterior thalamic radiation, CAB = cingulum angular bundle, CCG = cingulum cingulate gyrus, CST = corticospinal tract, ILF = inferior longitudinal fasciculus, SLFP = superior longitudinal fasciculus parietal, SLFT = superior longitudinal fasciculus temporal, UNC = uncinate fasciculus, lh = left hemisphere, rh = right hemisphere
