# Supplementary material for: Multimodal assessment of white matter tracts in amyotrophic lateral sclerosis
Source: PLoS One. 2017 Jun 2;12(6):e0178371. doi: 10.1371/journal.pone.0178371 (PMC5456080; doi:10.1371/journal.pone.0178371)
Supplement: S2 Table — The Pearson correlation coefficient is provided. (PDF) [file pone.0178371.s002.pdf]

S2 Table. Relationship between Clinical Status and Quantitative MRI for Individual Tracts. The Pearson correlation coefficient is provided**.**

		Disease Duration	ALSFRS-R	ALSFRS-R_{3M}	ΔALSFRS-R	Penn UMN score
**AD**						
	lh ATR	-0.14	-0.06	-0.14	-0.41*	-0.05
	lh CAB	0.20	-0.02	-0.11	-0.41*	0.37
	lh CCG	0.11	-0.46*	-0.44*	-0.16	-0.19
	lh SLFT	0.01	-0.11	-0.19	-0.41*	0.11
	lh UNC	0.29	-0.07	-0.17	-0.47*	0.04
	rh SLFP	-0.06	-0.10	-0.19	-0.46*	0.12
	rh UNC	0.10	-0.04	-0.15	-0.48*	-0.06
**RD**						
	FMINOR	0.00	-0.03	-0.12	-0.39*	-0.27
	lh ATR	-0.01	-0.14	-0.22	-0.43*	-0.18
	lh ILF	-0.04	-0.19	-0.27	-0.44*	0.20
	lh UNC	0.23	-0.27	-0.36	-0.52*	-0.05
	rh UNC	0.07	-0.05	-0.17	-0.52*	-0.25
**MD**						
	FMINOR	0.05	0.00	-0.09	-0.40*	-0.23
	lh ATR	-0.06	-0.12	-0.21	-0.46*	-0.15
	lh CAB	0.13	0.02	-0.08	-0.42*	0.11
	lh CST	0.12	-0.18	-0.26	-0.41*	0.19
	lh UNC	0.26	-0.20	-0.30	-0.52*	-0.02
	rh SLFP	-0.03	-0.05	-0.14	-0.39*	0.07
	rh UNC	0.08	-0.05	-0.17	-0.53*	-0.20
**FA**						
	lh ILF	0.14	0.14	0.23	0.49*	-0.28
	lh UNC	-0.03	0.38	0.43*	0.42*	0.11
	rh UNC	0.02	0.03	0.12	0.40*	0.35
**MTR**						
	FMAJOR	-0.07	-0.12	-0.19	-0.31	0.63*
	lh CAB	-0.58*	0.16	0.22	0.28	0.02
	lh CCG	-0.53*	0.03	0.04	0.02	-0.08
	rh UNC	-0.39	0.11	0.25	0.54*	-0.44
**R_2***						
	lh CST	-0.07	0.29	0.32	0.25	-0.44*
	lh ILF	-0.07	0.05	0.16	0.43*	-0.13
	lh SLFT	-0.55*	0.20	0.23	0.20	-0.03
	rh ATR	-0.20	0.09	0.23	0.56*	-0.40
	rh CST	0.34	0.04	0.11	0.28	-0.53*
	rh ILF	0.07	0.14	0.23	0.42*	-0.01

* p value < 0.05. Values indicate the Pearson correlation coefficient. AD = axial

diffusivity, RD = radial diffusivity, MD = mean diffusivity, FA = fractional anisotropy, MTR = magnetization transfer ratio, N = number of subjects, ALS = amyotrophic lateral sclerosis, FMAJOR = corpus callosum forceps major, FMINOR = corpus callosum forceps minor, ATR = anterior thalamic radiation, CAB = cingulum angular bundle, CCG = cingulum cingulate gyrus, CST = corticospinal tract, ILF = inferior longitudinal fasciculus, SLFP = superior longitudinal fasciculus parietal, SLFT = superior longitudinal fasciculus temporal, UNC = uncinate fasciculus, lh = left hemisphere, rh = right hemisphere, ALSFRS-R = revised ALS functional rating scale, ALSFRS-R_{3M} = ALSFRS-R 3 months follow-up, UMN = upper motor neuron
